# Cataract

**Published:** 1903-03-28

**Authors:** 


### CATARACT.

We have so long been in the habit of looking on senile cataract as merely one of the many degenerative lesions of old age, to avoid which seems scarcely to come within the scope of practical medicine, that it is with a pleasurable surprise one reads Dr. J. W. Jervey's[1] vigorous account of his new theory of the causation of this affection and notes the enthusiasm with which he upholds that in early cases the course of the disease may be arrested, if not completely cured, by operation. He commences by describing a case which came to his notice, and which apparently led him to think out his new theory. A man, 60 years of age, received a blow in the right eye 20 years previously, since which time his right pupil has been paralysed. Equatorial radial opacities are present in the lens cortex of the uninjured eye, but the lens of the right eye is clear. Adopting Schoen's view that all simple and senile cataracts commence as a capsulitis, Dr. Jervey goes on to suggest that this condition is set up through the irritation of a diseased iris constantly rubbing against the capsule. A portion of the posterior surface of the iris lies and moves in contact with the surface of the anterior capsule of the lens. In youth the iris is composed of soft, almost impalpable, tissue. The passage of this filmy substance across the surface of the capsule is not harmful. But let the character of its tissues become hardened or roughened, and there is an immediate plausible causation of capsulitis, for the iris never rests. It is not a coincidence, Dr. Jervey holds, that in the majority of cases of cataract it is impossible to obtain full dilatation of the pupil under the influence of mydriatics. It is to be accounted for by the thickening and stiffening of the iris and not, as is commonly suggested, by atrophic conditions. The causes of these changes in the iris are various. There is, first and foremost, pre-eminent in the causation of senile cataract, arterio-sclerosis; there are rheumatism and gout, the circulatory changes of Bright's disease and of diabetes and the results of other chronic inflammations of the intra-ocular structures. Any of these may be the cause of changes which eventuate in cataract. But the abnormal, rough, thickened iris is the medium through which the cataract is started. It is not all cataracts but only a majority that are formed in this way—there are certain forms due to other causes, *e g.* trauma. With regard to treatment, Dr. Jervey says: Interrupt the iris and prevent its activity in those eyes — and they are many—in which iridian friction is irritating the anterior capsule and leading to opacification, and the cataractous process will be arrested. To do this iridectomy is necessary. This operation he thinks will only hasten the process if it be done so that the lens is injured during the operation, and to avoid this the utmost skill and delicacy will be required. All that is wanted is a tiny piece snipped out of the pupillary border of the iris sufficient to break the continuity of the sphincter. This will result in partial dilatation of the pupil and cessation of hippus. If, in the course of the operation, the forceps rub upon the surface of the lens, even with the iris tissue intervening, the purpose of the effort may be thwarted, and instead of arrest there may be increase of progress of the opacities. Should this happen no great harm will have been done, and nothing at all to endanger the success of a subsequent extraction. He does not advocate sphincterectomy, as he calls it, to the exclusion of all other efforts; constitutional treatment is still important, and glasses should invariably be adjusted to keep pace with any refractive changes that may occur. The measures indicated must be resorted to early—in the very commencement of opacity formation.

[1] New York Medical Record, Feb. 28.

